# Bax/Bcl-2 Cascade Is Regulated by the EGFR Pathway: Therapeutic Targeting of Non-Small Cell Lung Cancer

**DOI:** 10.3389/fonc.2022.869672

**Published:** 2022-03-25

**Authors:** Manzar Alam, Shoaib Alam, Anas Shamsi, Mohd Adnan, Abdelbaset Mohamed Elasbali, Waleed Abu Al-Soud, Mousa Alreshidi, Yousef MohammedRabaa Hawsawi, Anitha Tippana, Visweswara Rao Pasupuleti, Md. Imtaiyaz Hassan

**Affiliations:** ^1^ Centre for Interdisciplinary Research in Basic Sciences, Jamia Millia Islamia, Jamia Nagar, India; ^2^ Department of Biotechnology, Jamia Millia Islamia, Jamia Nagar, India; ^3^ Department of Biology, College of Science, University of Hail, Hail, Saudi Arabia; ^4^ Department of Clinical Laboratory Science, College of Applied Sciences-Qurayyat, Jouf University, Sakaka, Saudi Arabia; ^5^ Department of Clinical Laboratory Sciences, Faculty of Applied Medical Sciences, Jouf University, Sakaka, Saudi Arabia; ^6^ Health Sciences Research Unit, Jouf University, Sakaka, Saudi Arabia; ^7^ Molecular Diagnostics and Personalized Therapeutics Unit, University of Hail, Hail, Saudi Arabia; ^8^ Research Center, King Faisal Specialist Hospital and Research Center, Jeddah, Saudi Arabia; ^9^ Regional Agricultural Research Station, Acharya N. G. Ranga Agricultural University (ANGRAU), Tirupati, India; ^10^ Department of Biomedical Sciences and Therapeutics, Faculty of Medicine & Health Sciences, University Malaysia Sabah, Kota Kinabalu, Malaysia; ^11^ Department of Biochemistry, Faculty of Medicine and Health Sciences, Abdurrab University, Pekanbaru, Indonesia; ^12^ Centre for International Collaboration and Research, Reva University, Rukmini Knowledge Park, Bangalore, India

**Keywords:** NSCLC, B cell lymphoma 2, Bax, apoptosis, signaling, targeted therapy

## Abstract

Non-small cell lung carcinoma (NSCLC) comprises 80%–85% of lung cancer cases. EGFR is involved in several cancer developments, including NSCLC. The EGFR pathway regulates the Bax/Bcl-2 cascade in NSCLC. Increasing understanding of the molecular mechanisms of fundamental tumor progression has guided the development of numerous antitumor drugs. The development and improvement of rationally planned inhibitors and agents targeting particular cellular and biological pathways in cancer have been signified as a most important paradigm shift in the strategy to treat and manage lung cancer. Newer approaches and novel chemotherapeutic agents are required to accompany present cancer therapies for improving efficiency. Using natural products as a drug with an effective delivery system may benefit therapeutics. Naturally originated compounds such as phytochemicals provide crucial sources for novel agents/drugs and resources for tumor therapy. Applying the small-molecule inhibitors (SMIs)/phytochemicals has led to potent preclinical discoveries in various human tumor preclinical models, including lung cancer. In this review, we summarize recent information on the molecular mechanisms of the Bax/Bcl-2 cascade and EGFR pathway in NSCLC and target them for therapeutic implications. We further described the therapeutic potential of Bax/Bcl-2/EGFR SMIs, mainly those with more potent and selectivity, including gefitinib, EGCG, ABT-737, thymoquinone, quercetin, and venetoclax. In addition, we explained the targeting EGFR pathway and ongoing *in vitro* and *in vivo* and clinical investigations in NSCLC. Exploration of such inhibitors facilitates the future treatment and management of NSCLC.

## 1 Introduction

Among all cancer deaths, lung cancer mortality is very common, estimated up to 1.7 million per year worldwide (1). There are two histological subtypes: non-small cell lung carcinoma (NSCLC) and small cell lung carcinoma (SCLC). NSCLC comprises around 80% to 85% of all lung cancer cases. Tobacco smoking is the root cause of NSCLC which comprises around 80% of cases in the United States and other countries where smoking is common (2). Although the most common etiology behind NSCLC and SCLC is smoking, lung adenocarcinoma (LUAD) is mostly associated with non-smokers. Non-smoker LUAD is commonly found in East Asian women with environmental exposure and genetic reasons. According to the recent document, the standard 5-year survival rate for NSCLC is barely 16% (3). Over half of lung tumor cases are diagnosed following metastasis, for which the mean survival time is about 8 months. Among lung tumor bone metastasis, the majority common target organ is the vertebral column, which causes more severe effects on patients’ recovery rate and life worth (4, 5).

EGFR overexpression has been involved in cancers, including NSCLC (6). EGFR is one of the most generally mutated genes in NSCLC (7). EGFR is associated with several human malignancies (8). Around 10%–30% of NSCLC patients have activating mutations in EGFR (9, 10). Increased EGFR protein and mRNA expressions are linked with poor prognosis, tumor growth, metastasis, and resistance to chemotherapy (11). EGFR activation is linked with proliferation, metastasis, apoptosis inhibition, and radio-chemotherapy resistance in cancer (12). Aberrant activation of the EGFR pathway axis has been found to play a major role in cancer (13). A study reported MAPK1 amplification in an erlotinib-resistant EGFR-mutant NSCLC (14). The histological transformation to SCLC in EGFR mutant-NSCLC patients with acquired EGFR TKI resistance. Similarly, aberrant induction of the EGFR–STAT-3–Bcl-xL signaling axis has been observed to fuel cancer progression (13, 15, 16). Bcl-2 and Bcl-xL are Akt/EGFR downstream pathway proteins which were reported to be conscientious to drug resistance in several tumors, such as SCLC (17, 18).

EGFR has been considered an important target for NSCLC therapeutics (19). Numerous studies have documented the mechanisms engaged in the progression of AR to EGFR TKI, which may be potential therapeutic strategies (20, 21). The inhibition of EGFR could be a potential clinical strategy for inhibiting and overcoming EMT-linked acquired drug resistance that affords inspiration for the clinical trial of combined EGFR and FGFR blockage in EGFR-mutated NSCLCs (21, 22). Targeted therapy opens new dynamics in lung cancer management by identifying altered target genes. Targeting EGFR in the patients with stimulating mutations showed initial and considerable success in the clinic (23, 24). However, the inhibition of EGFR leads to upregulation of pro-apoptotic proteins and, lastly, results in apoptosis by activating the intrinsic apoptotic pathway (25, 26). Earlier reports (27, 28) demonstrated the important function of Bcl-2 in the resistance of NSCLC for EGFR TKIs. It validated that growth inhibition is induced by the treatment activation of caspase-3 and Bax, supposedly by EGFR, ERKs, and MMP-2 downregulation (29). To develop effective therapies against lung cancer, it is very important to understand its biology at the molecular level (30). In the current study, we presented recent information on the molecular mechanisms of the Bax/Bcl-2 cascade-mediated EGFR pathway in NSCLC and its therapeutic implications along with clinical investigations that facilitate the treatment and management of several cancers, including NSCLC.

## 2 Regulation of Bax in NSCLC

**Figure 1:**
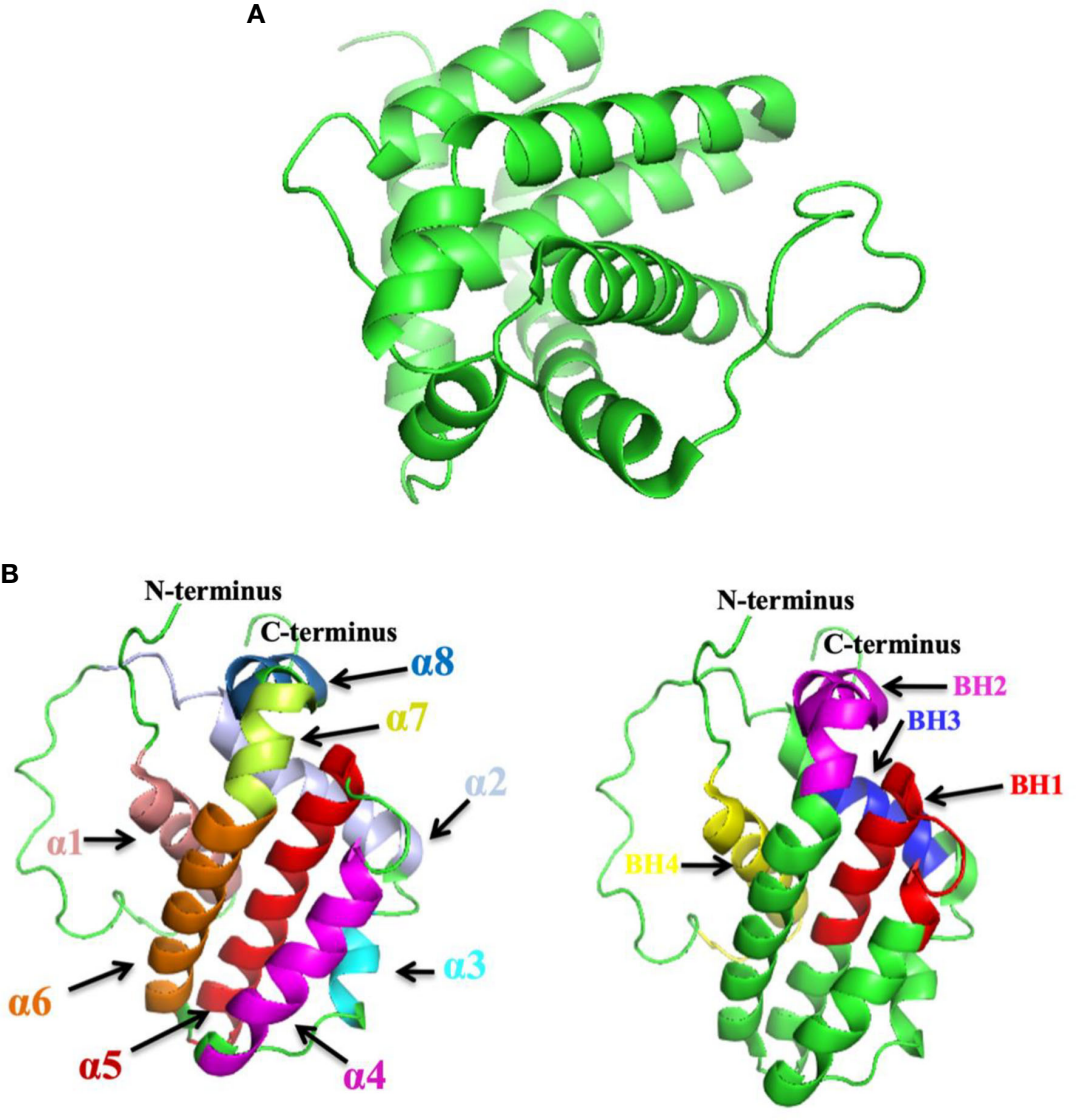
**(A)** The tertiary structure of Bax (PDB ID: 1F16). **(B)** The tertiary structure of Bcl-2; highlighted sections indicate α-helices (α1–α8) and BH1-4 domains. However, the left panel denotes the Bcl-2 structure with α1–α8 with several colors. The right panel denotes the Bcl-2 structure with the BH1-4 domains (PDB ID: 1G5M). (Structure was drawn by PyMol, 40).

Bax is a pro-apoptotic protein that plays a pivotal role in controlling apoptosis. It is generally present in the cytoplasm, which is heterodimerized with anti-apoptotic proteins. When a cell is exposed to an apoptotic stimulus, Bax protein is translocated to the mitochondria (31). The increased expression of Bax mediates in early apoptosis (32). Bcl-2 family members share at least one of four types of homology, namely, BH1, BH2, BH3, and BH4 (33). Their structure can be homodimers or heterodimers having nine α helices and a hydrophobic α-helix embedded in the core with a transmembrane terminal C attached at the mitochondrial lining (33–35). The activation of Bax can be initiated by various abiotic factors such as heat, pH change, and stress conditions (36, 37). p53 upregulates Bax in the stress environment as a stimulus response, further activating downstream target genes like Bax (38). The *Bax* gene was first reported as one of the important pro-apoptotic Bcl-2 family proteins (39). The tertiary structure of Bax is exhibited in **Figure 1A**.

The human Bax gene is present in chromosome 19q and consists of six exons and four variants (41). Mutations and alterations in the coding regions and promoters of the *Bax* gene have been detected for affecting the protein expression and function in a variety of malignancies (42, 43). Mutations in the *Bax* gene are very frequent, resulting in loss of the tumor-suppressor function and resistance to apoptosis and chemotherapy (44, 45). The single-nucleotide polymorphism (SNP) of the *Bax* gene, i.e., at -125 nucleotides, a G to A transition, from the beginning of transcription, and at -248 nucleotides from the translation initiation, has been recognized in cancer. This SNP was linked with altered mRNA/protein expression associated with cancer development and chemoresistance (46, 47). An elevated risk of HNSCC in patients having the AA genotype of G(-248)A SNP was reported (42). Hence, Bax is an important gene in oral cancer development. The effects of promoter methylation/SNP, mutations of the exons, and reduced expression of Bax were associated with oral cancer development (48).

A low expression of Bax has been drastically linked with NSCLC patients (49) and poor prognosis in NSCLC patients (50). A decreased expression of Bax and p53 was comparatively resistant to cisplatin and decreased apoptosis in lung cancer cells (51). Radio-resistant NSCLC exhibits little box/Bak activation compared with radiosensitive NSCLCs (52). A low Bax protein expression was demonstrated for contributing to oral cancer progression (53, 54). A low expression of Bax is associated with decreased apoptosis, advanced-stage neoplasms, poor prognosis, cancer progression, and resistance to chemotherapy in colorectal cancer (55, 56). The regulation of Bax-induced apoptosis in NSCLC is incompletely understood.

## 3 Regulation of Bcl-2 in NSCLC

B cell lymphoma 2 (Bcl-2) is an anti-apoptotic protein encoded by the *Bcl-2* gene in the human genome specified as an oncogene (57). It was recognized for its involvement in t(14;18) chromosomal translocations detected in non-Hodgkin’s lymphomas (57). Bcl-2 is ~250 kb in length and made up of three (03) exons and two (02) promoters (58), exon 1 and exon 2 encoding all four BH domains, whereas exon 3 encodes the TM domain that connects the protein to intracellular membranes (59, 60). It was the primary protein to be recognized among Bcl-2 family proteins. There are two isoforms of Bcl-2: Bcl-2α and Bcl-2β. Hence, Bcl-2α is anti-apoptotic (61). The Bcl-2 protein has a common genetic region in the BH domain; it includes up to four conserved BH domains (62, 63). Bcl-2 (239 amino acids) contains four domains, namely, BH1, BH2, BH3, and BH4 (64, 65). Additionally, these domains compose BH4 domain (10–30) residues, BH3 domain (93–107) residues, BH1 domain (136–155) residues, and BH2 domain (187–202) residues (64). Bcl-2 explains a tertiary structure **(** **Figure 1B** **)** enclosing two hydrophobic α-helices (Hα5 and Hα6) surrounded by amphipathic α-helices (40, 66). The *Bcl-2* gene activates *via* a chromosomal translocation mechanism in many human tumors (67, 68). Bcl-2 inhibits apoptosis *via* inhibiting the liberation of cyt-*c*, thus blocking the activation of caspases that stimulate apoptosis (69). Bcl-2 binds with BH3 domains of Bax and inhibits their functioning (70, 71).

Bcl-2 promotes cell survival and viability and regulates mitochondrial dynamics like fusion and fission. An increased Bcl-2 expression has been observed in NSCLC (72). The expression of Bcl-2 and mutated p53 might be highly resistant to cisplatin and have low susceptibility for apoptosis in lung cancer cells (51). Bcl-2 has induced cancer growth and resistance to chemotherapeutics in xenograft models of NSCLC (73, 74). Bcl-2 is considered a good prognostic marker in NSCLC (75, 76) and a constructive prognostic biomarker in LUSC (77). The evaluation of Bcl-2 expression by tumors could provide predictive data on the clinical manners of NSCLC (78). An excessive expression of Bcl-2 and a suppressed expression of Bax lead to homeostatic disbalance of cells, subsequently causing cancer. However, according to a recent study, 76% of SCLC is caused by the overexpression of Bcl-2 (79, 80). Bcl-2 is a critical player in imparting resistance to cancer cells (81, 82). Increased Bcl-2 expression is linked with advanced-stage neoplasms and poor differentiation (83) and is found to resist chemotherapy in many cancers (56). A high expression of Bcl-2 protein was found in many drug-resistant cancer cells (53, 84). However, Bcl-2 transcript cleavage induces cell death and impairs cell survival (85, 86). The upregulation of Bcl-2 defends drug-mediated cells from apoptosis (87). Bcl-2 participates in a fundamental function in cancer growth, angiogenesis, and tumor vascular density (88). Therefore, Bcl-2 is involved in NSCLC.

## 4 Regulation of EGFR in NSCLC

**Figure 2:**
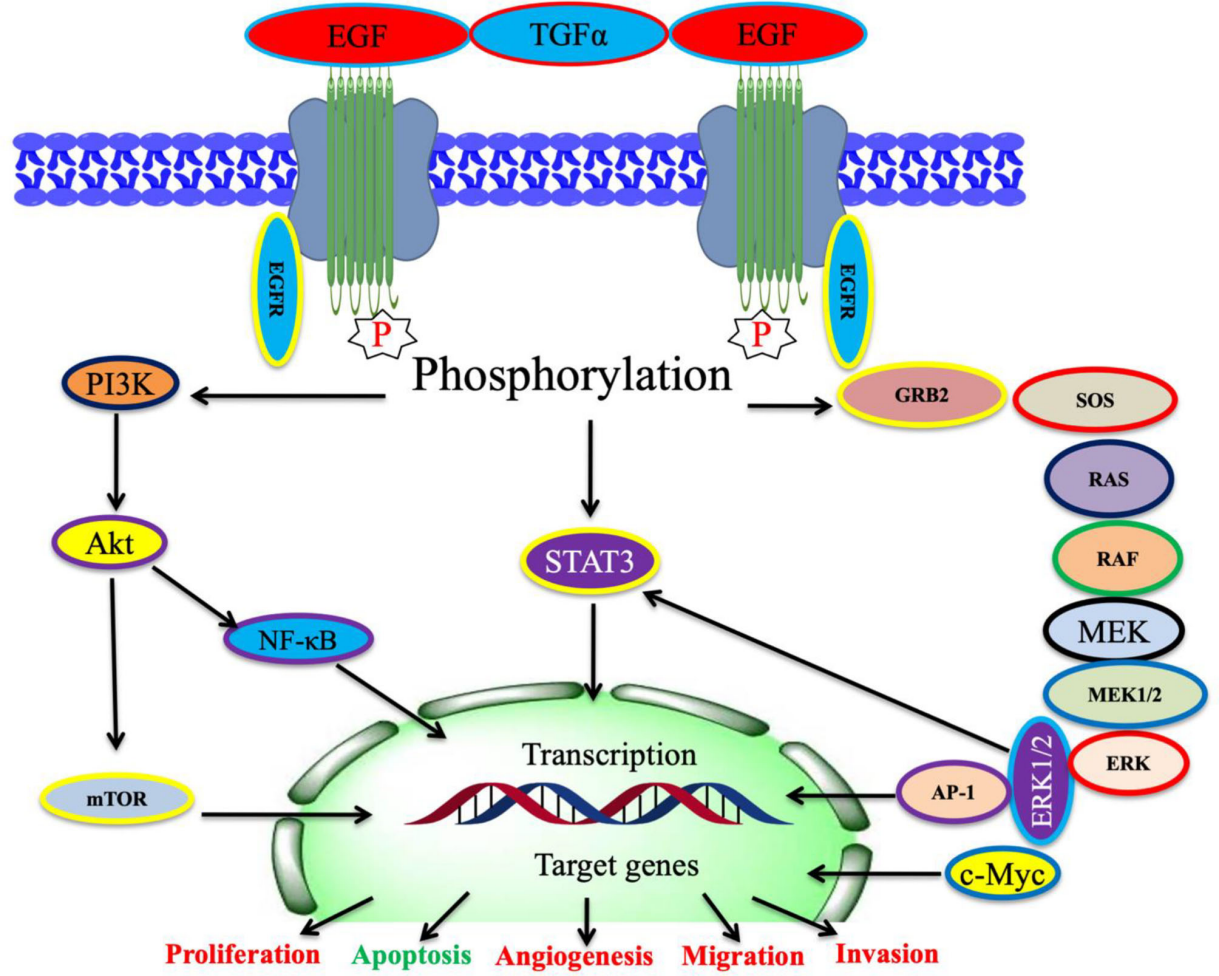
The EGFR receptor and its signaling cascade involved in cancer progression. (This figure is adapted from Ref 97–99).

EGFR contains an extracellular EGF-attaching domain, a transmembrane domain, and a cytoplasmic domain (89). EGFR is a transmembrane cell-surface receptor. The tyrosine kinase (TK) receptor is commonly activated in epithelial tumors (90). The corresponding mRNA is encoded from 28 exons spanning approximately 190,000 (nucleotides) on chromosome 7p12. It belongs to the ErbB family of receptor tyrosine kinases that also includes ErbB2 (HER-2 or Neu), ErbB3 (HER-3), and ErbB4 (HER-4) (91). The activated EGFR causes the activation of several pathways, including ERK and Stat-3 pathways (92). ERK and Stat-3 are the important signaling molecules under EGFR (93). EGFR activates the PI3K-Akt, STAT, and MAPK pathways, eventually leading to enhanced cell proliferation, survival, and migration (26, 94–96) (**Figure 2**). EGFR overexpression has been involved in multiple cancers, including NSCLC (6). EGFR is one of the most generally mutated genes in NSCLC (7). Around 10%–30% of NSCLC patients have activating mutations in EGFR (9, 10). Major scientific and clinical studies have proved that alleles of patients with NSCLC revealed mutations in *KRAS* and *EGFR* genes, which demonstrate their function in tumor development and progression (100, 101). KRAS and EGFR mutations occur mutually and have symbiotic relations where *KRAS* mutations may bestow resistance to EGFR inhibitors (102, 103).

Several studies have explained mutations in the EGFR gene (6, 104). EGFR mutations are frequently oncogenic; specifically, they trigger the EGFR pathway in the lack of ligand and endorse cell survival and anti-apoptotic signals (6, 105). EGFR, TK type I receptors, and its gene are situated at the short arm of human chromosome 7 (106). In EGFR, 28 exons form a protein, which is dispensed on the cell membrane of several epithelial cells, wherever it attaches to EGF or heparin-binding EGF and controls cell growth (107). By comparison, exon 20 insertions and exon 18-point alterations/mutations are less general than exon 19 deletions and exon 21 L858R substitutions in EGFR mutations in NSCLC (108, 109). The regulation and activation of EGFR and downstream genes initiate apoptosis, angiogenesis, and proliferation (110). Hence, the most frequent are first short in-frame deletions about the LREA motif of exon 19 (~45%–50% mutations) and 2nd point mutations (CTG to CGG) in exon 21, which affects the substitution of leucine *via* arginine on codon 858, L858R (~45%–50% mutations) (111, 112). These alterations/mutations are more common in NSCLC (111–113). The histological transformation to SCLC in EGFR mutant-NSCLC patients with acquired EGFR TKI resistance. Numerous studies have documented the mechanisms engaged in the progression of AR to EGFR TKI, which may be potential therapeutic strategies (20, 21).

More EGFR protein and mRNA expressions are linked with poor prognosis, more tumor growth, metastasis, and resistance to chemotherapy (11). EGFR plays a major role in various human malignancies (8). EGFR activation is linked with the malignant phenotype, blockage of apoptosis, increased proliferation, metastasis, and resistance to radio-chemotherapy (12). Aberrant activation of the EGFR pathway axis was found to play a major role in HNSCC (13). EGFR is associated with oral cancer development and chemoresistance (93, 114). EGFR activation has been detected in oral cancer (8, 115). EGFR is linked with inhibition of apoptosis and resistance to chemotherapy in various tumors (116, 117). Numerous signaling molecules lie downstream of EGFR involved in cancer development (93).

## 5 Targeting the EGFR Pathway in NSCLC

Targeted therapy opens new dynamics in lung cancer management by identifying altered target genes. According to the biological function of diverse forms of EGFR in NSCLC, EGFR-targeted therapy is divided into two parts: “EGFR mutant targeted therapy” and “wt EGFR targeted therapy.” Among NSCLC patients, ~10%–30% have lung cancers with EGFR mutations (118). EGFR mutant targeted therapy targets the main oncogene linked with tumorigenesis and is important for cancer maintenance. However, this therapy displays a remarkable comeback in clinical TKI treatment through induction of apoptosis. Similarly, above 70% of NSCLC patients by wt EGFR treated with TKIs are getting “wt EGFR targeted therapy.” Therefore, it targets a protein not openly connected to cancer initiation but more liable to cell growth. This therapy is far less efficient in the clinical phase. It is normally linked with growth arrest and stable disease (SD) (118, 119).

The EGFR mutational summary of NSCLCs is a good predictor of reaction for therapy with the extremely efficient TKIs (120, 121). TKIs have been considered proficient drugs in NSCLC and have provided brilliantly targeted drugs (122). TKIs targeting EGFR were checked in clinical trials approved by the FDA (121, 123). Multiple agents/drugs targeting EGFR have appeared, including gefitinib, erlotinib, panitumumab, and cetuximab (124–126). Among the approved EGFR-TKIs, gefitinib, lapatinib, erlotinib, and icotinib are categorized as first-generation and afatinib, neratinib, and dacomitinib are categorized as second-generation EGFR inhibitors. The third-generation EGFR inhibitors include olmutinib, almonertinib, and osimertinib (127). In addition, vandetanib, brigatinib, and pyrotinib are categorized as multi-kinase inhibitors, because of their inhibitory actions against kinases, excluding the EGFR (128).

**Figure 3:**
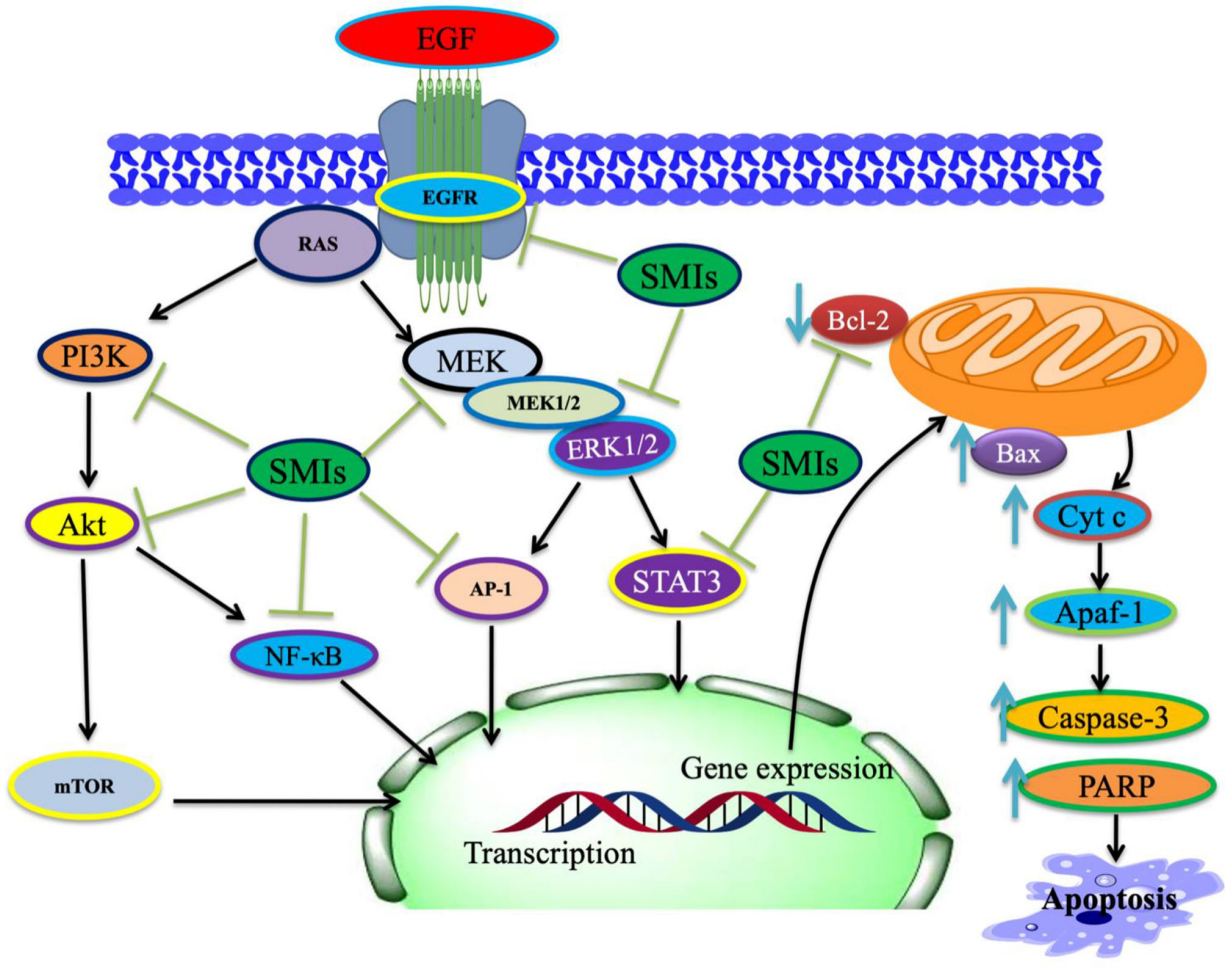
The proposed possible mechanism of small molecule inhibitors (SMIs)/phytochemicals in EGFR-mediated pathways along with Bax/Bcl-2 cascade in NSCLC cells. SMIs, small-molecule inhibitors. (This figure is adapted from Ref 97, 133). This figure was drawn by ChemBioDraw.

Targeting EGFR signaling represents a new strategy for personalized medicine in NSCLC. Targeting EGFR in the patients with stimulating mutations showed initial and considerable success in the clinic (23, 24). Some were developed to target EGFR, including TKIs and BRAF inhibitors (129, 130). Inhibiting EGFR-mediated activation of the downstream pathway, EGFR TKIs can influence the cellular level of apoptotic-linked proteins, primarily the pro-apoptotic consequence of EGFR targeting (131, 132). EGFR may be a potential clinical strategy for inhibiting EMT-linked acquired drug resistance and EGFR blockage in EGFR-mutated NSCLCs (21, 22). However, the inhibition of EGFR leads to upregulation of pro-apoptotic proteins and, lastly, results in apoptosis by activating the intrinsic apoptotic pathway (25, 26) (**Figure 3**). Earlier reports (27, 28) demonstrated the important function of Bcl-2 in the resistance of NSCLC for EGFR TKIs. It validated that growth inhibition is induced by the treatment activation of caspase-3 and Bax, supposedly by EGFR, ERK, and MMP-2 downregulation (29, 134).

## 6 Therapeutic Target of Bax/Bcl-2 Cascade and EGFR-Mediated NSCLC by Phytochemicals/Small-Molecule Inhibitors

Scientific data exhibit that phytochemicals have considerable anticancer potential. Roughly 50% of approved antitumor drugs originated from natural produces or derived from that place (135). However, these phytochemicals were tested for antitumor efficiency at *in vitro* and *in vivo* levels. They possess balancing and overlapping mechanisms for slowing down the carcinogenic procedure *via* scavenging free radicals (136), repressing growth and proliferation (137), and reducing angiogenesis and invasiveness of cancer cells (138). They exert an extensive and complex range of acts on several molecular targets as well as signal transduction pathways such as membrane receptors (139), kinases (140), downstream tumor-activator or -suppressor proteins (141), and transcriptional factors (142). Several phytochemicals/SMIs have been exhibited as potential therapeutics for NSCLC. The inhibitors of Bcl-2 are used for the therapeutic targeting of several tumors **(** **Table 1** **)**. Some of the incredible antitumor phytochemicals/SMIs in this regard are explained in the present study. Here, we have discussed selective phytochemicals/SMIs including gefitinib, EGCG, ABT-737, thymoquinone, quercetin, and venetoclax that inhibit and target EGFR pathways in NSCLC (**Figure 3**).

**Table 1:** The inhibitors of Bcl-2 are used for the therapeutic targeting of several tumors.

Agents	Structure	IC_50_ for Bcl-2 (μM)	Clinical status	References
Gefitinib	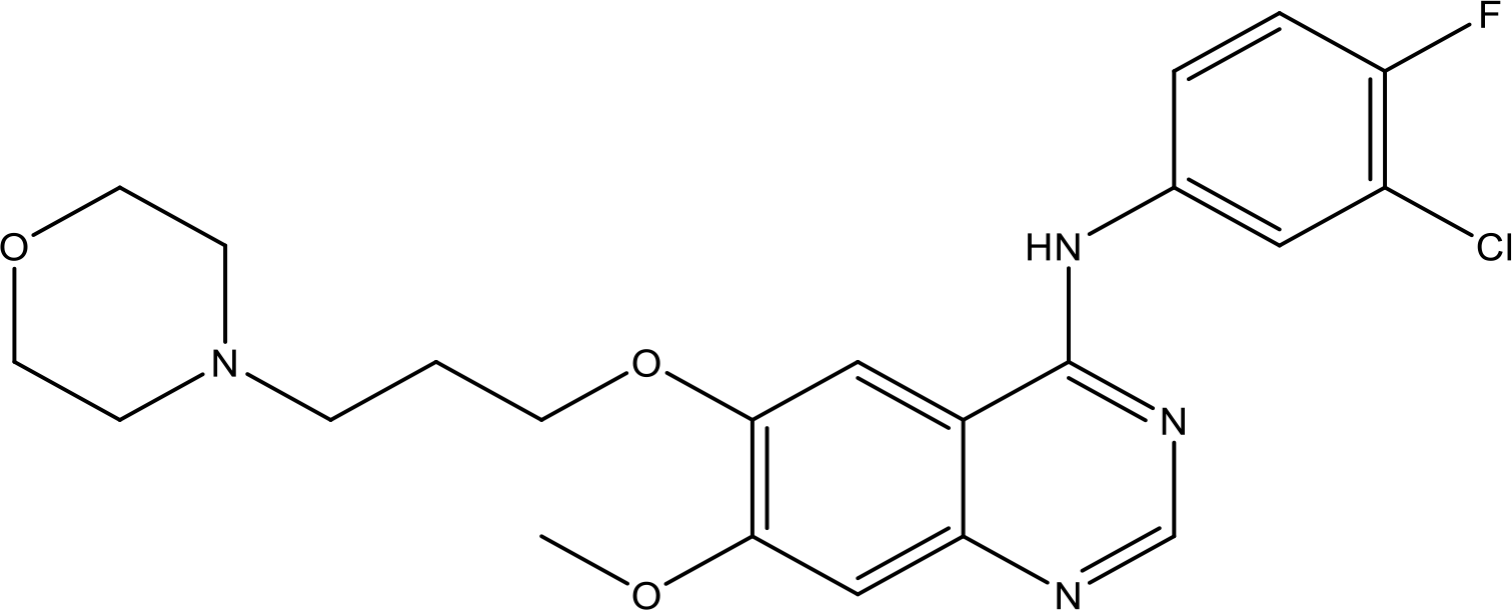	17.12	Phase 2	(143)
Thymoquinone	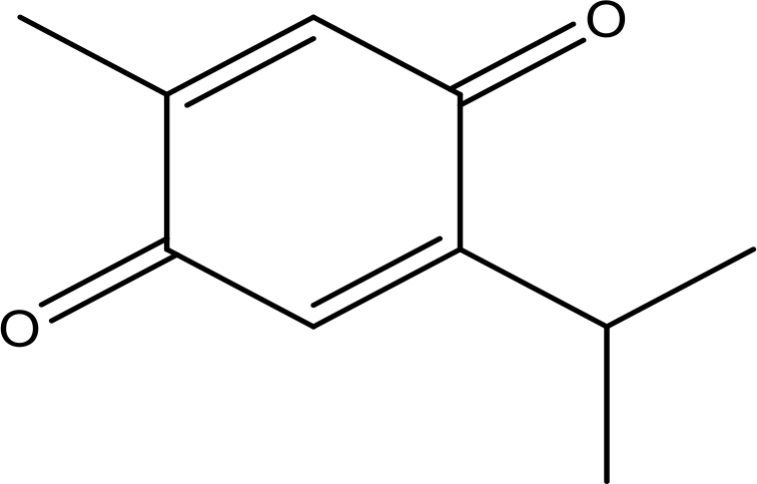	45.78	Phase 2	(144)
Quercetin	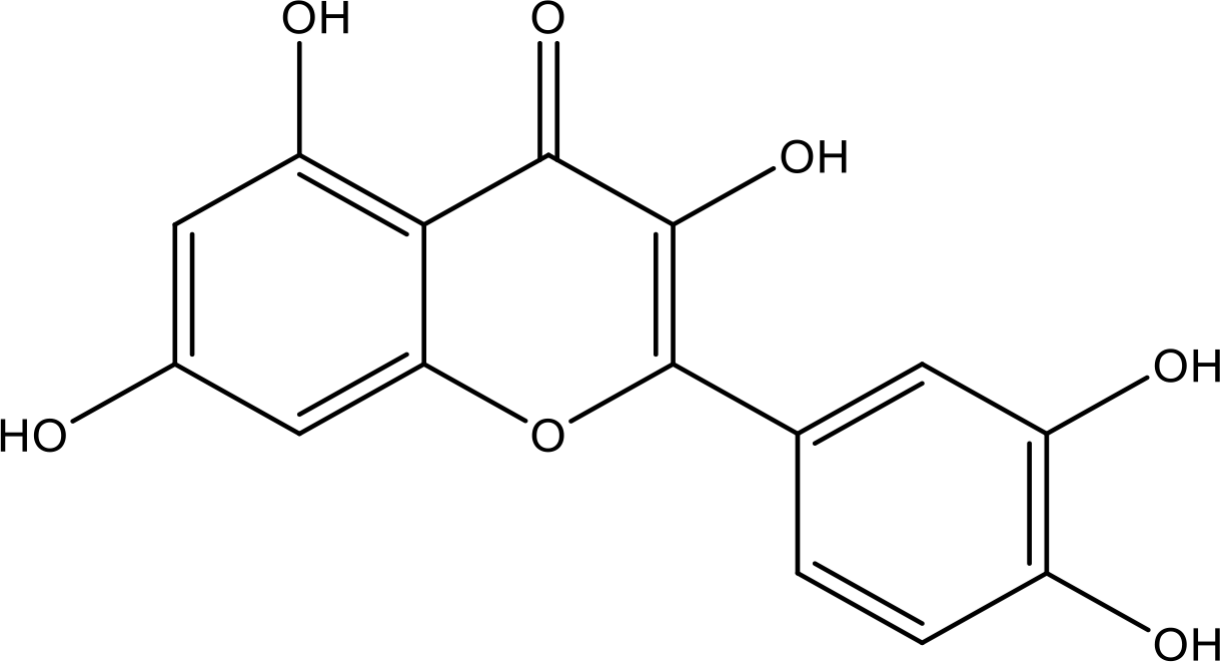	35.69	Phase 2	(144)
EGCG	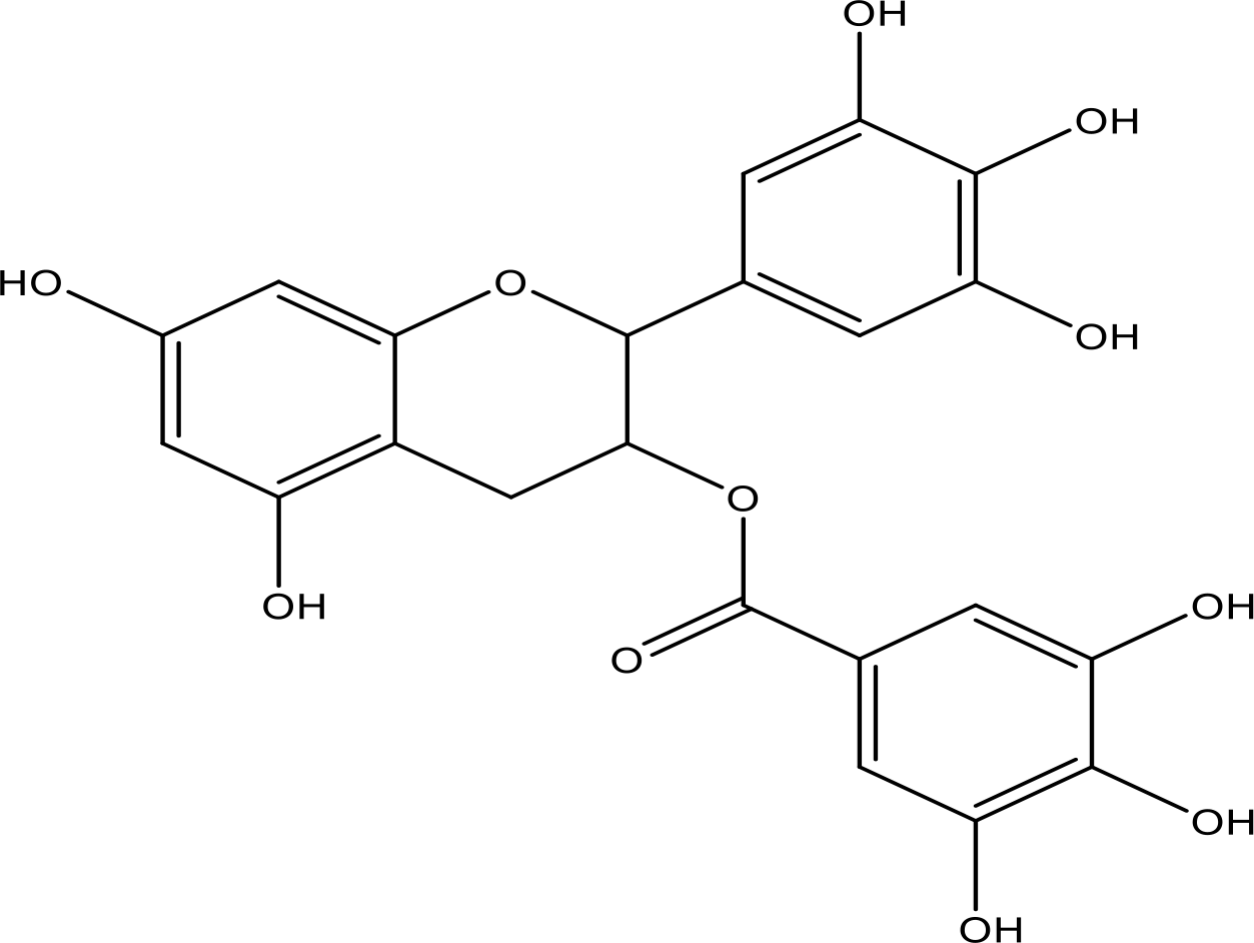	0.45	Phase 1/2	(145, 146)
ABT-737	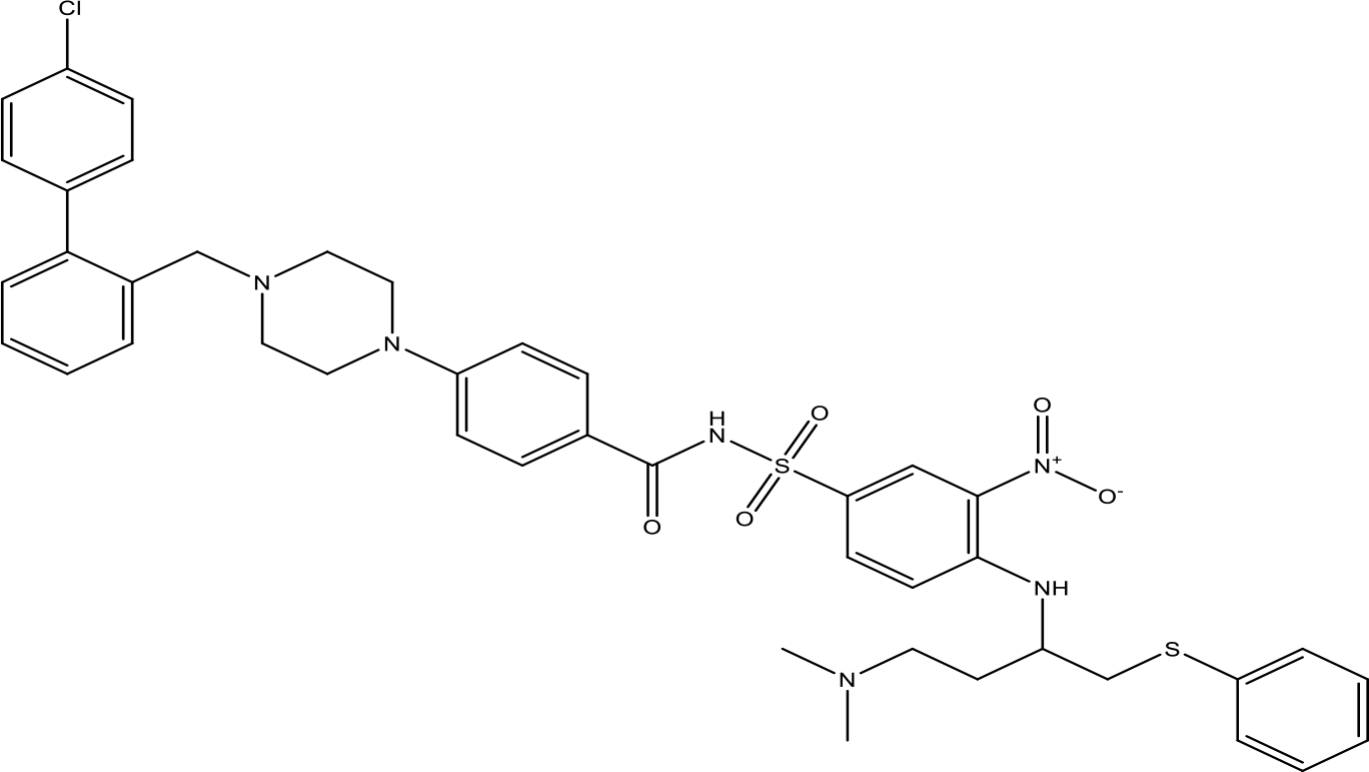	0.12	Phase 1/2	(147, 148)
ABT-263	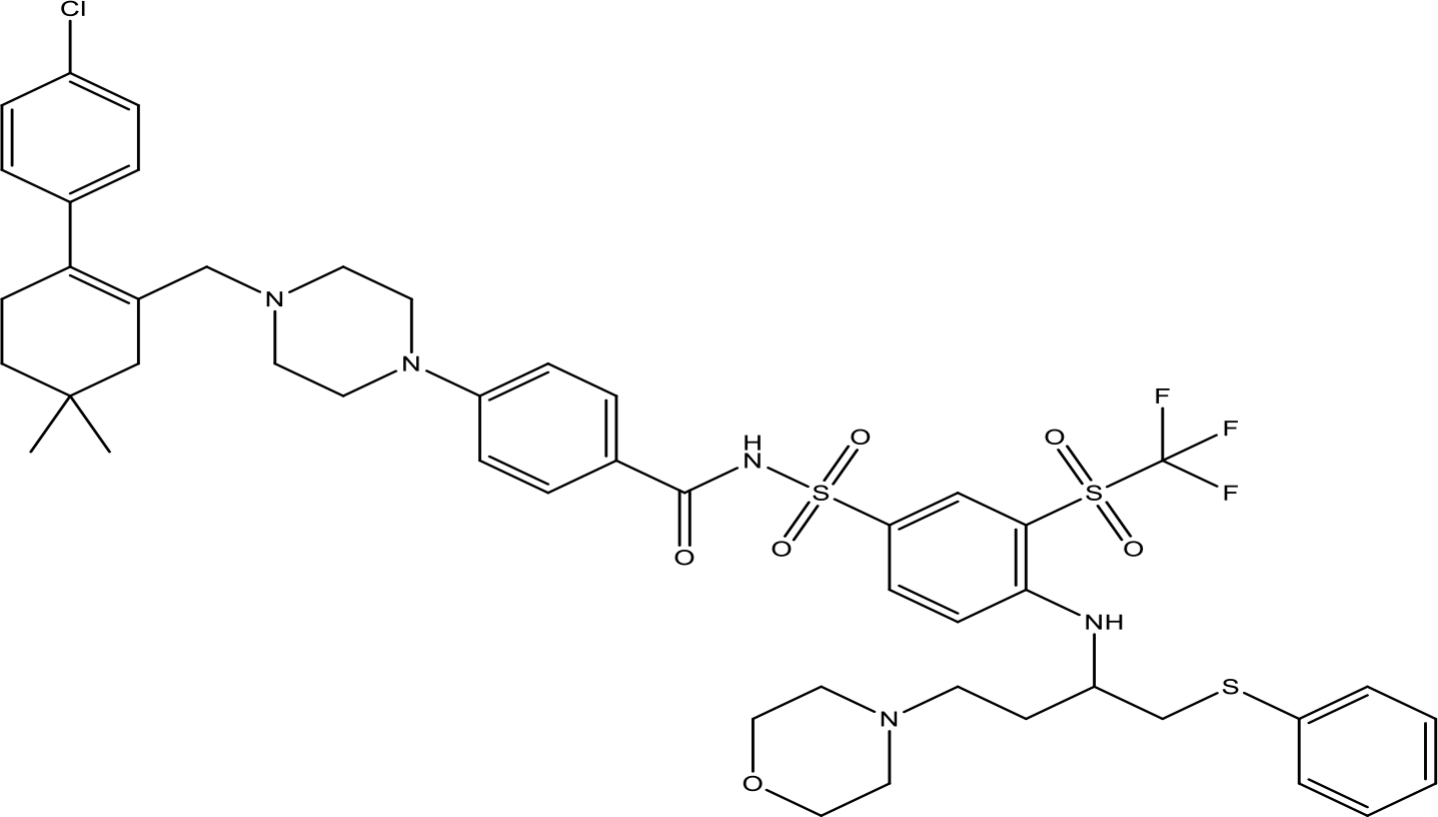	NA	Phase 1/2	(149, 150)
ABT-199	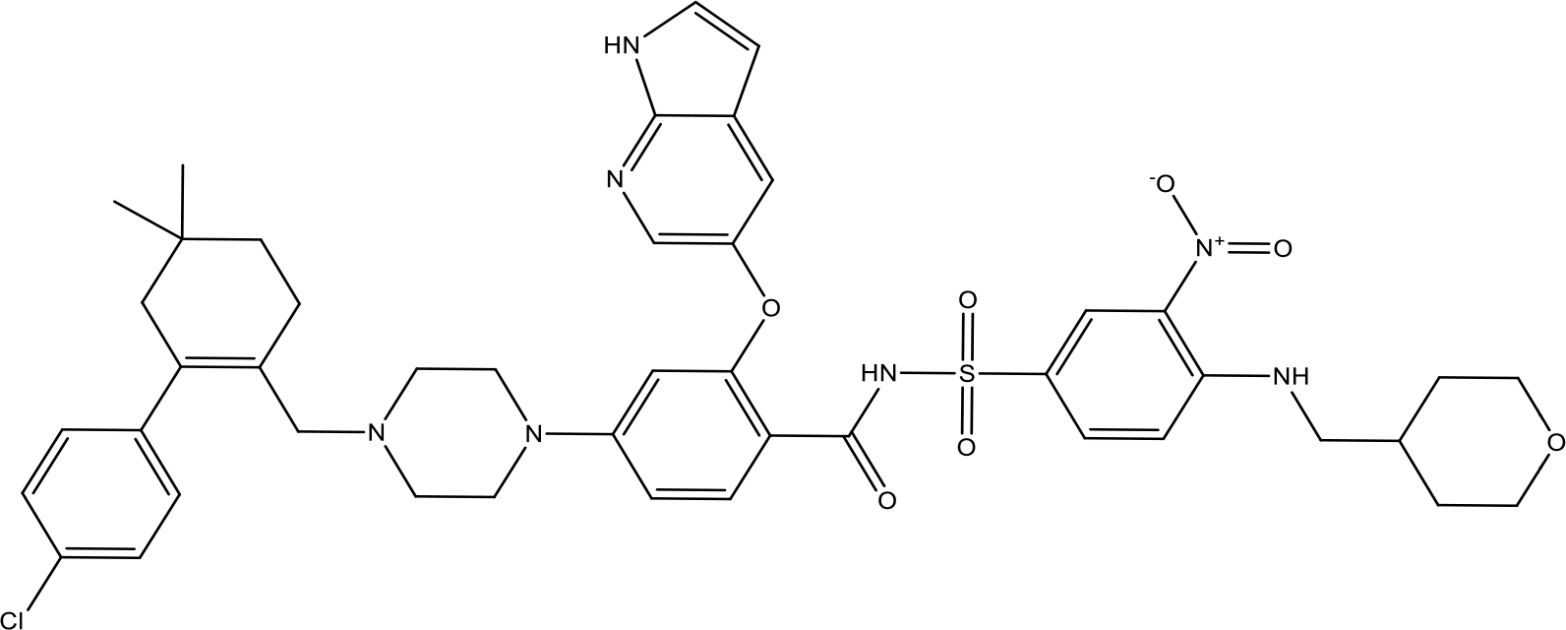	0.1	Approved for use in CLL	(151)
TW-37	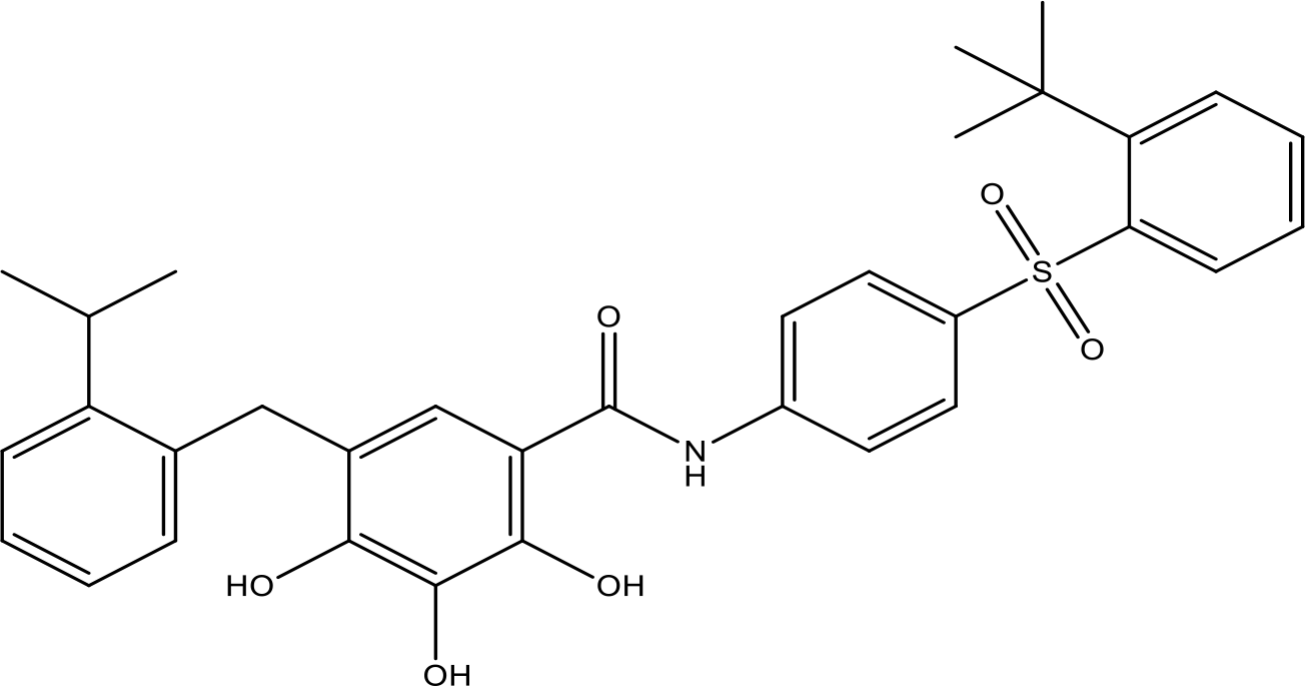	NA	Phase 1/2	(152, 153)
Gossypol	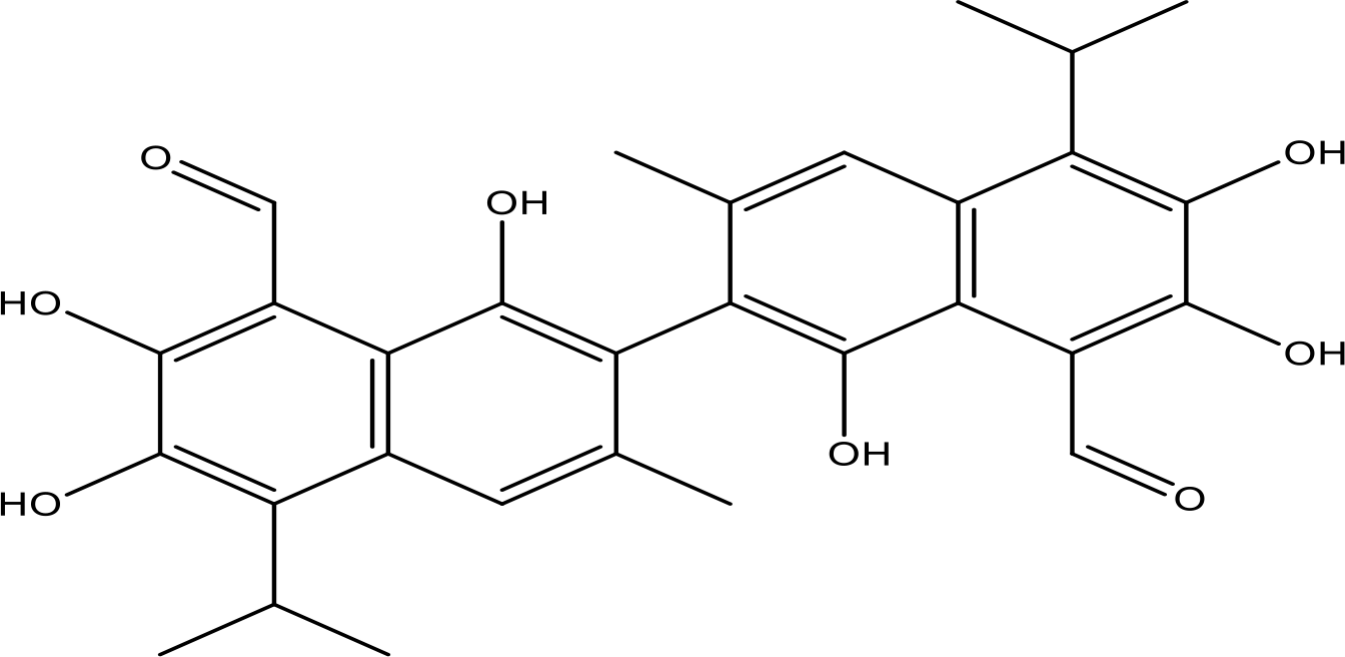	0.28-10	Phase 1/2	(154)
GX15-070 (obatoclax)	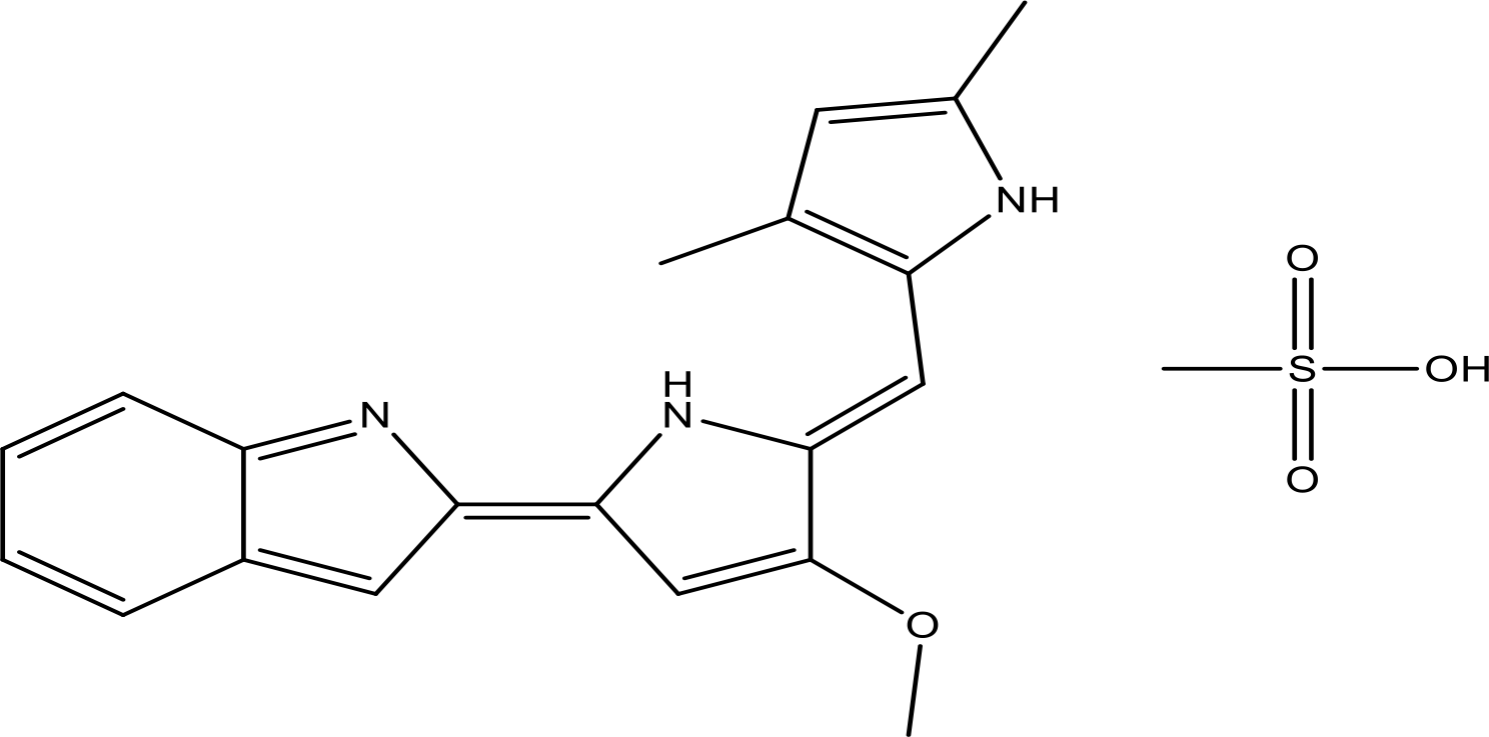	NA	Phase 1	(155, 156)
HA14-1	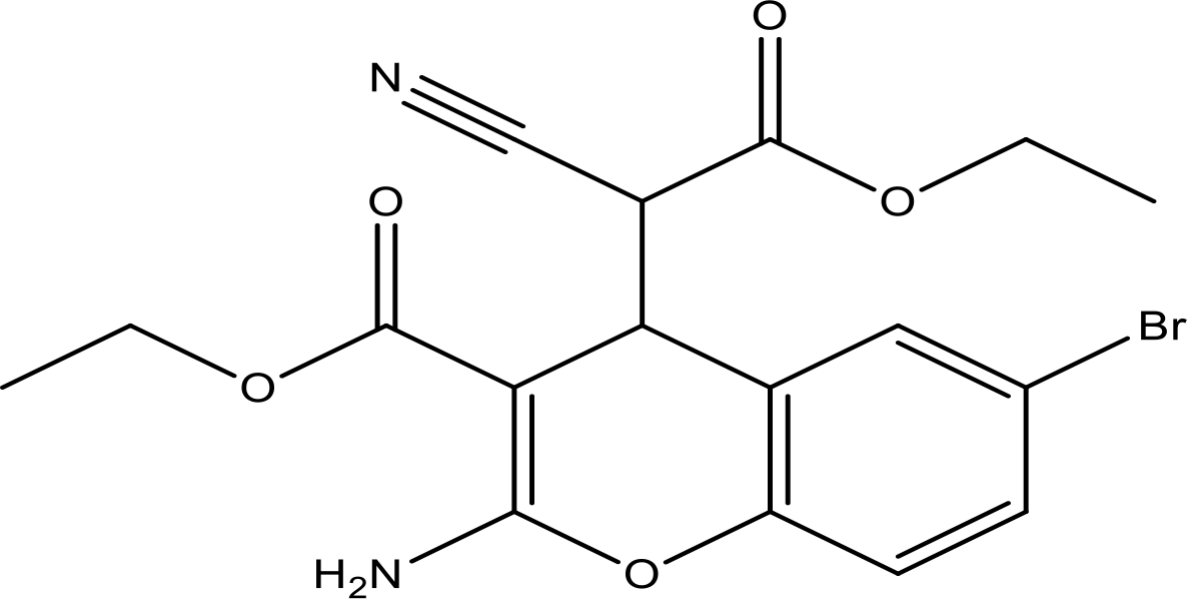	~9	Preclinical	(155, 157)
Chelerythrine	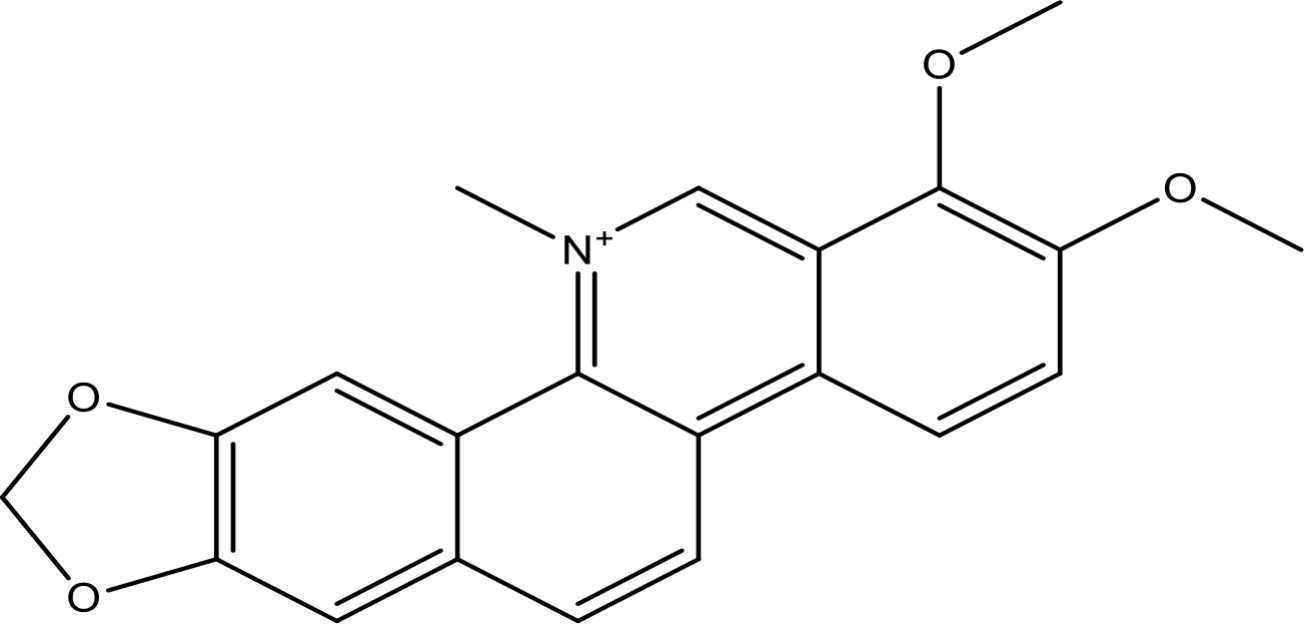	~10		(158)
S55746	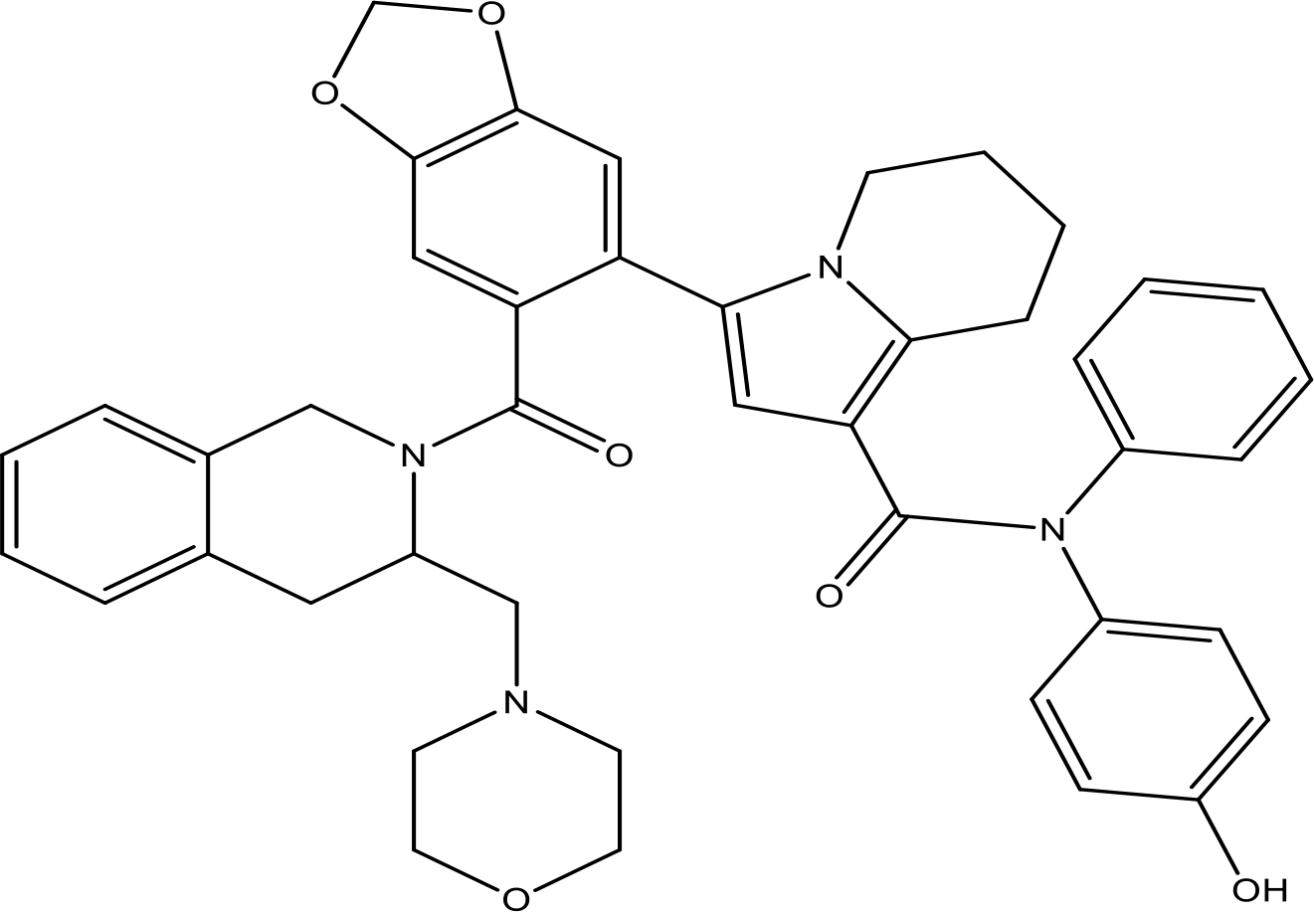	NA	Phase 1	(159)

### 6.1 Mechanism of Gefitinib in NSCLC

Gefitinib showed potent activity in NSCLC (160). IDEAL 1 and 2 trials have been designed to further examine the efficiency and safety of two diverse gefitinib doses in patients with pretreated NSCLC (161, 162). This test validated that gefitinib is dynamic in greatly pretreated NSCLC patients, with a reaction rate of 11.8% and symptom enhancement in 43% of patients in the 250-mg arm (162). A randomized phase II trial compared gefitinib with docetaxel for advanced NSCLC patients (163, 164). Six phase III trials estimated the force on survival of erlotinib/gefitinib alone or in combination with therapy in metastatic or advanced NSCLC patients (165–167). Hence, the *in vitro* actions of gefitinib against susceptible and resistant cancer cells have been evaluated in numerous reports (168, 169). A study (170) examined the result of gefitinib on the cell proliferation of NSCLC cell lines utilizing the MTS test and colony formation tests. However, the results exhibited IC_50_ values of 4–42 μM.

#### 6.1.1 Effect of Gefitinib on Bax/Bcl-2 Cascade

Gefitinib-mediated apoptosis is increased *via* accumulation of the BH3 mimetic ABT-737 (171). It stimulates apoptosis by activation of Baxin cancer cells. However, it stimulates G1 arrest and apoptosis *via* regulating p21 and p27 and the activation of Bax in GBC cells (172). The less regulation of Bcl-2 *via* RNAi in gefitinib-resistant H1975 cells with T790M mutation increased the results of gefitinib and can offer a new therapeutic approach for NSCLC treatment (28). Gefitinib could be most efficient in NSCLC patients (173). It repressed the expression of Bcl-2 and Bcl-xL, rendering HCC prone to cell death (174). Gefitinib combined with cinobufotalin obstructs viability and assists the apoptosis of A549 cells, showing that the combined therapy may be a potential novel treatment for lung cancer patients resistant to gefitinib (143). This is a new therapy for the treatment of NSCLC.

#### 6.1.2 Effect of Gefitinib on the EGFR-Mediated Pathway

Gefitinib has been the initial SMI of EGFR identified for clinical application (175). Gefitinib is a TKI that treats NSCLC patients whose cancers have particular EGFR mutations (176). It was approved for cancer treatment in May 2003 (176) and approved for metastatic EGFR mutation-positive NSCLC in July 2015 (177). Gefitinib blocks EGF-mediated EGFR autophosphorylation in many EGFR-expressing cancer cells (178). The kinase inhibitory action of gefitinib, along with eight of the approved kinase inhibitors, has been assessed (179, 180). The kinase inhibitory actions have been evaluated against 310 kinases utilizing an activity-based kinase test, and it has been observed that gefitinib especially prevents EGFR and its mutants. In addition to the inhibition of EGFR, the results exhibited gefitinib’s capability to inhibit the serine/threonine kinases at IC_50_ = 50 and 90 nmol/l, respectively (RICK and GAK). However, gefitinib might have cellular option modes of activity. The cellular IC_50_ of gefitinib against several EGFR mutants has been established (181). A study reported the relationship of growth inhibition on the ERK1/2 and Akt activation and in response to the EGFR pathway (170). Hence, a study displays that the accumulation of a BH3 mimetic drastically increases the killing of NSCLC cells *via* EGFR TKI gefitinib (171).

### 6.2 Mechanism of EGCG in NSCLC

EGCG is the most plentiful polyphenol in green tea (182, 183). The therapeutic results of EGCG were identified against various tumors (184–186). EGCG was examined in many tumor cells and a few clinical trials with minimum information on its efficiency in lung cancer. EGCG damages growth in SCLC cells. However, a variable result on the limited number of NSCLC cells was checked (187, 188). EGCG is a promising antioxidant with various beneficial results in oxidative stress-mediated disorders (189). EGCG contributes to blocking NO and H_2_O_2_ production in human skin (190). EGCG might powerfully inhibit oxidative stress-induced protein tyrosine nitration *via* oxidative stress in blood platelets (191), and antioxidants may progress the function of mitochondria (192). However, EGCG is a well-recognized antioxidant and quenches ROS, supporting oxidative DNA break, cancer endorsement, and mutagenesis that lead to anticancer effects (193).

#### 6.2.1 Effect of EGCG on the Bax/Bcl-2 Cascade

EGCG induced apoptosis by enhancing the Bax and cleaved caspase-3 expression and dropping the Bcl-xL expression in cancer cells (194). EGCG diminished the regulation of Bcl-2 and Bcl-xL (40, 195). EGCG mediated apoptosis *via* an intrinsic pathway by caspase-9 activation in PC3 cells (196) and MCF-7 cells (197). However, EGCG-mediated cell death of tumor cells has been correlated with the decline in the expression of Bcl-2. EGCG has displayed the induction of apoptosis by enhancing caspase-3, caspase-9, BAD, cyt-*c*, PTEN, SMAC, and Fas and repressed Bcl-2, Bcl-xL, and c-Myc in cancer cells (198, 199). Green tea improved the apoptotic efficiency in cancer (200). It has anticancer effects, which enhanced Bax, Bak, and PUMA and reduced Bcl-xL and Bcl-2 that activate caspases-9, inducing apoptosis in cancers (97, 201, 202). Recently, the interaction of EGCG with p53 disrupts p53 with its regulatory E3 ligase MDM2 and reduces the ubiquitination of p53 through MDM2. Since EGCG interrupts the binding of p53 for its regulator MDM2, p53 is stabilized through blocking p53 ubiquitination as well as degradation (203). EGCG was recognized from a library of about 2,295 phytochemicals like an inhibitor of p53 with MDM2 interaction (204).

#### 6.2.2 Effect of EGCG on the EGFR-Mediated Pathway

EGCG prevents several signal transduction pathways in tumor cells. Hence, EGCG blocked proliferation in various NSCLC cells (205). EGCG induces apoptosis by the mitochondrial pathway and inhibits EGFR, ERK, and STAT3 signaling in HNSCC (145, 146). EGCG prevented STAT3 activation and downregulation of the target genes, including Bcl-2, Bcl-xL, Mcl-1, cyclin D1, and VEGF (206). However, EGCG blocked NF-κB, ERK1/2, and Akt-induced pathways, thereby modifying the Bcl-2 family protein ratio that activates caspases in tumor cells (207–209). Inhibition of c-Jun N-terminal kinase *via* EGCG induced apoptosis in OSCC cells (201, 210). EGCG might inhibit p-Akt/p-mTOR expression through PTEN to control the PI3K/Akt pathway (211, 212). EGCG exposure noticeably reduced EGF-mediated EGFR, ERK1/2, and Akt activation. However, long-term EGCG treatment prevented the total and membranous expression of EGFR and noticeably attenuated EGFR nuclear localization and cyclin D1 expression, showing that EGCG treatment repressed EGFR transactivation. However, inhibition of the EGFR pathway could partially contribute to the antitumor action of EGCG in lung cancer (213).

The wnt/β-catenin pathway was encouraged in lung tumor stem cells. Hence, EGCG decreased lung tumor stem cells’ action by reducing lung tumor stem cell markers, blocking tumorsphere formation, reducing cell proliferation, and promoting cell death (214). EGCG was observed for blocking angiogenesis and diminishing xenograft cancer growth *via* inhibiting IGF-1 by repressing HIF-1a and VEGF in A549 cells (215–217). EGCG blocked HGF-induced cell growth and invasion *via* repression of HGF/c-Met signaling in SCC VII/SF cells, whereas it blocked xenograft cancer survival *in vivo* by increasing cell death (218). Numerous *in vitro* studies explained the anticancer effect and potential mechanisms of EGCG on tumor cells. The combination treatment blocked the EGFR pathway and reduced the p-EGFR, p-ERK, and p-Akt expression *in vitro* and *in vivo*. EGCG and cDDP have exhibited a potential therapeutic effect in NSCLC patients (219). Nano-EGCG can prevent lung cancer cell proliferation, invasion, and migration *via* the activation of AMPK pathways. However, this mechanism of nano-EGCG recommends its application in lung cancer treatment and prevention (220).

### 6.3 Mechanism of ABT-737 in NSCLC

ABT-737 is an SMI designed to especially block anti-apoptotic Bcl-2 proteins (221, 222). This molecule, a BH3 mimetic, attaches with more affinity to Bcl-2, and Bcl-xL stimulates apoptosis (221). ABT-737 may improve the radiosensitivity of a variety of solid cancers. However, the radiosensitizing effect of ABT-737 has been examined in NSCLC (223). Despite its potential results in *in-vitro* studies and tests on animal models, ABT-737 was approved for clinical trials due to unfavorable pharmacological features such as thrombocytopenia (222, 224–227). The clinical significance of Bax is largely reported in several studies. Several Bax-targeted anticancer drugs are approved for medical use, for example, ABT-737 (228). This class of drugs has proved their potential in reversing the resistance effect. Therefore, there is the bigger necessity to develop cost-effective gene-targeted combinatorial drugs from natural products (66, 228).

#### 6.3.1 Effect of ABT-737 on Bax/Bcl-2 Cascade

Abbott developed ABT-737, a novel inhibitor of Bc-l-2, in the last decade which was expected to target Bcl-2, Bcl-2-X, and Bcl-2-w to show promising results at the research stage. These were developed as BH3-targetable small-molecule inhibitors (SMI). They enhance the apoptotic effects in SCLC (229). ABT-737 is a potent inhibitor of Bcl-2, Bcl-w, and Bcl-xL (222). It is a promising SMI of anti-apoptotic proteins such as Bcl-2 in HNSCC (147). ABT-737 activates caspase-3, which leads to apoptosis. ABT-737 upregulates the Noxa expression. Noxa by small interfering RNA attenuates cell death (147). It attaches with a very high affinity with Bcl-2. Bcl-2 is hindered because of ABT-737 into its hydrophobic groove, and this binding dislocates any bound BH3-enclosing proteins (148). ABT-737 induced the caspase-3 activation and cleavage of PARP that stimulated apoptosis. Glioblastoma cells’ large quantities of Bax protein are prebound with Bcl-2, which are acutely liberated *via* ABT-737 treatment. However, the highly “addicted” cells become Bax-neutralizing Bcl-2 proteins (230). ABT-737 holds huge promise, as it passionately attaches the pro-survival proteins similar to Bcl-2 and stimulates Bax/Bak-dependent destruction. BT-737 regulated Bax/Bak-induced apoptosis (231).

#### 6.3.2 Effect of ABT-737 on EGFR-Mediated Pathway

Several studies exhibited the potential effect of ABT-737 on signaling molecules. BIM polymorphism is strongly linked to a poor clinical reaction for EGFR TKIs in EGFR-mutant NSCLC patients; hence, BH3-mimetic ABT-737 returns BIM functionality EGFR-TKI sensitivity (232). ABT-737 drastically increases erlotinib-mediated cell death, and more strong responses for EGFR inhibitors in lung tumor patients harbor EGFR kinase domain mutations (233). Bcl-2 antagonist ABT-737 slights the apoptotic threshold to chemotherapeutic drugs *via* the PI3K/Akt signaling inhibition in cancer cells. Inhibition of Bcl-2 and Bcl-xL increases Akt/PI3K inhibition-induced apoptosis in cancer cells (234, 235). The p53 and Akt pathways were examined to be associated with the effect of ABT-737 and naringenin in gastric cells (236). The PI3K/Akt inhibitor BEZ235 with ABT-737 regulates ovarian cancer cell apoptosis (237). However, targeting the Akt/PI3K/mTOR and/or ERK/MAPK pathways may disturb the imbalance between anti-apoptotic and pro-apoptotic partners that might constitute an important approach for sensitizing cancer cells for ABT-737 (238). Inactivation of ERK1/2 with ABT-737 enhanced the BIM expression that induced apoptosis in oral cancer cells. Targeting the ERK1/2-bim pathway *via* BH3-mimetic ABT-737 is an optional therapeutic approach for oral cancer (239). The phosphorylation of Bcl-2 on Ser-70 through JNK and paclitaxel synergizes by ABT-737 and reinstates paclitaxel sensitivity in breast cancer cells (240). ABT-737 recovered the radiation sensitivity of HeLa cells, thereby stimulating cell death, and showed that ABT-737 reduced HeLa cell proliferation and activated JNK (c-Jun), which resulted in more regulation of BIM (241). The sensitizing results were detected when ABT-737 was combined with sorafenib that effectively repressed levels of STAT3. They suggested that targeting STAT3 in combination with inducers of the apoptosis pathway may be a potential novel strategy for treating tumor cells (242).

### 6.4 Mechanism of Thymoquinone in NSCLC

There are numerous bioactive ingredients extracted from *Nigella sativa (black seeds)*, which show anticancer activities by modulation of cell-cycle pathways, but thymoquinone (TQ) is regarded as the most potent anticancer bioactive compound found in black seeds (243). Others compounds are dithymoquinone (DTQ), thymohydroquinone (THQ), and thymol (THY) (244, 245). *N. sativa* has numerous valuable constituents that effectively treat various diseases (246). TQ has been identified to exert antioxidative, anti-inflammatory, and anticancer effects (247, 248). TQ plays an effective role in cancer treatment by inducing apoptosis or suppressing the expression of carcinogens (249–251). TQ displays inhibitory results on numerous processes of NSCLC, including apoptosis proliferation, migration, and angiogenesis (252). TQ blocked the growth and decreased expression of cyclin D1 in A549 cells (NSCLC) (253, 254). However, TQ synergistically augments conventional medicine prevention of NCI-H460 cells (252). It displays a therapeutic role in lung cancer (255–257). TQ blocks cell proliferation, stimulates apoptosis, and obstructs xenograft cancers’ *in vivo* growth of numerous tumor cells, including lungs (247, 252).

TQ is a powerful anti-carcinogenic and anti-mutagenic mediator (258, 259). Aqueous and alcohol extracts of *N. sativa* were effectual in inactivating MCF-7 breast cancer cells (258, 260). *In-silico* screening is an excellent approach used to screen potential anticancer compounds. It also helps our body soak up the medicine quickly when taken in petite dosages. Therefore, it indicates a potential aspect of combinatorial therapy (261, 262). Although there are several constituents of *N. sativa* which played a beneficial role in disease management, its chief constituent TQ has proved its active role in cancer prevention. TQ imparts antioxidant effects in animal models (263–265). Cancer as a disease relies on multiple factors like modification in genetic pathways (266, 267). Black seed preparation also helps to reduce the toxicity and side effects of anticancer synthetic drugs (268, 269). TQ showed a protective role in oxidative stress conditions when administered orally by inducing free radical generation (270–272). Few researchers suggested that oral administration of TQ alleviates quinine reductase and glutathione transferase (273, 274). Therefore, TQ can be used as a drug to counter the toxicity of liver carcinogens (275, 276). This property of TQ attributes to its protective role in balancing the toxicity of chemotherapy-based treatments (277). TQ has therapeutic implications in health and cancer management by genetic cascade modulations. It functions through the activation of the cancer suppression gene (278).

#### 6.4.1 Effect of Thymoquinone on Bax/Bcl-2 Cascade

Alterations in the normal process of cell death (apoptosis) increase the chances of cell survival and thus lead to cancer development and progression. Bax/Bcl-2 cascade is critical to inducing apoptosis, as already reviewed. Still, there is scope to determine its role in other important cancer-related pathways like modulation of Bax-Bcl-2 cascade (279). TQ phytosomes stimulated apoptosis at 4.31 ± 2.21 µM by caspase-3 activation and generation of ROS, besides gathering cells on G2-M and pre-G1 in A549 cells (280). TQ increased apoptosis *via* enhancing the Bax/Bcl-2 ratio and more regulating the expression of p53 in A549 cells (281). TQ stimulates the tumoricidal action of NK against lung tumor cells *via* more regulating pro-apoptotic genes and less anti-apoptotic genes (282, 283). TQ considerably diminished the viability of HCT116 cells in a concentration- and time-dependent way. However, treatment of cells with TQ-mediated cell death has been linked with the more regulation of Bax and downregulation of Bcl-2 and Bcl-xL (284). TQ stimulated caspase-9,-7, and -3 and activated PARP. TQ modulates the action of the Bax/Bcl-2 cascade. Here, TQ was found to downregulate the expression of Bcl-2, thus inducing apoptosis (285). This study also showed that TQ elevates ROS expression, which leads to a decrease in MMP, also known as DNA laddering, and the subsequent release of cyt-*c* (285). TQ upregulates the Bax/Bcl-2 ratio, thus inhibiting downstream caspases in the hepatic ischemia–reperfusion injury (I/R) model (286). TQ also increases the Bax/Bcl-2 ratio while upregulating the expression of Bax. In one of the studies, TQ was found to elevate the expression of Bax in Hl-60 cells, thus activating upstream caspase 8 results in the release of cytochrome c (287). These observations regarding TQ inspire the scientific community to develop gene target-based combinatorial therapies, where TQ can play a significant role in blocking the expression of Bcl-2 and counter toxicity of chemotherapy drugs.

#### 6.4.2 Effect of Thymoquinone on EGFR-Mediated Pathway

TQ is associated with multiple pathways; treatment with TQ reduced the phosphorylation of JAK2, Src kinase, and EGFR. TQ stimulated apoptosis in HCT116 cells *via* inhibiting the STAT3 pathway by inhibition of JAK2- and Src-induced phosphorylation of EGFR-TK (253, 284, 288). TQ attenuated the STAT3 expression target gene products, including c-Myc, survivin, and cyclin-D1 and -D2, and increased p27 and p21 (284). TQ might target multiple kinases, such as PI3K, MAPK, JAK/STAT, PLK1, and tyrosine kinase, in diverse cancer cells as well as animal models. However, inhibiting the action of kinases or repressing their expression may be among the mechanisms of TQ antitumor action. Targeting kinases with TQ, which is a molecularapproach for tumor therapeutics. (288). It illustrated the capability for suppressing the ERK1/2 pathway, which blocked the invasion and migration of A549 cells (254), and the therapeutic promise of TQ as an anti-metastatic drug in lung cancer treatment. Hence, subcutaneous doses of the TQ-I3M combination repressed the lung tumor metastasis and decreased tumor growth by the inhibition of the NF-κB/Akt/mTOR pathway in the xenograft model (253). It shows significant anticancer activities *via* upregulation of PTEN during transcription. It is well-known that PTEN played a role in inducing p53 expression and inhibits the Akt pathway (267). Apart from this, TQ also modulates various genetic pathways. It also inhibits NF-ĸB activation, which results in the downregulation of inflammatory genes. It upregulates miR34a and downregulates Rac1 expression (278).

### 6.5 Mechanism of Quercetin in NSCLC

Quercetin (Qu) is a flavonoid found abundantly in fruits (apple) and vegetables (onions), citrus foods, and tea. It has excellent antioxidant properties (289). It shows anticancer properties like growth factor suppression, apoptosis induction, and anti-proliferative actions (290). When treating A549 and H1975 cells for 24 h using the vehicle of Qu, there was no sign of altered viability compared to control (291). Qu suppresses the wound closure and invasive and migratory abilities of NSCLC cells at low concentrations (10–50 µM), indicating its anti-proliferative properties (292). Qu notably stimulated the apoptosis of cancer cells. It demonstrates antitumor roles both *in vivo* and *in vitro* (293). Apart from good results both *in vitro* and *in vivo*, some clinical studies have also evaluated the antitumor therapeutic results of Qu in ovarian cancer (294). In both *in vitro* and *in vivo* cancer studies, Qu was said to have a beneficial effect against prostate cancer (295).

This antioxidant effect of Qu can be attributed to its protective nature against the toxicity of drugs (296, 297). Studies were conducted to access its apoptosis induction potential and chemosensitivity, and the results were found to be appreciable and show the tumor inhibitory actions of Qu (298–300). Due to its antioxidant properties, it can also be used as a nutritional supplement in human health management. Several studies proved that it protects from the harmful effects of free radicals caused by smoking (296). Qu has excellent modulation properties toward inflammatory agents. It inhibits the core inflammatory enzyme COX (301–305). In one such study conducted by Cruz-Correa *et al*., combination doses of curcumin (480 mg) and Qu (20 mg) were orally administered to familial adenomatous polyposis (FAP) patients for 6 months three times a day. The result shows that this combination treatment reduces the size of rectal adenomas with no side effects (306). Qu inhibits hexavalent chromium (Cr[VI]) and shows a chemical carcinogen-induced cell transformation such as cell visibility loss, ROS generation, and microRNA-21 (miR-21) elevation in human colon cancer Caco-2-cells (307, 308).

#### 6.5.1 Effect of Quercetin on Bax/Bcl-2 Cascade

Qu inhibits cell proliferation, stimulates apoptosis, and functions as an antioxidant. It may modulate apoptosis by Bcl-2 family proteins that downregulate Bcl-xL and Bcl-2 and upregulate Bax and Bad (309, 310). However, Qu modulates the expression of the Bax/Bcl-2 cascade and thus mediates apoptosis (311–313). It stimulates apoptosis in caspase-3-dependent signaling *via* blocking Cox-2 expression and controls Bcl-2 and Bax expression. It might be a promising and potent agent that can be safely utilized in leukemia therapy (314). The results recommended that NSCLC H-520 cells with Qu enhanced the cisplatin-mediated apoptosis. However, this has been accompanied by downregulation of Bcl-2 and Bcl-xL and upregulation of Bax. Qu acts as an efficient chemo-sensitizer in the chemotherapy of lung tumor through controlling the expression of several apoptosis-linked genes (315). TQ and Qu drastically decrease the expression of Bcl-2 and induce Bax, indicative of sensitizing NSCLC cells stimulating apoptosis (144). Bcl-2 and Bcl-xL protein expressions were significantly decreased, and Bax and caspase-3 were increased treated by Qu (312).

#### 6.5.2 Effect of Quercetin on the EGFR-Mediated Pathway

The role of Qu-mediated molecular regulation in repressing NSCLC metastasis recommends that it has promising therapeutic functions for metastatic NSCLC (292). Qu is an effectual inhibitor for managing NSCLC harboring the EGFR C797S mutation. However, Qu displayed promising cytotoxic results on NSCLCs harboring the EGFR C797S mutation *via* preventing AXL and stimulating cell death (316). It mimics the interfaces of ATP in the active location of RTKs (EGFR, FGFR1, and c-Met) that lead to the prevention of C RTK overexpression (317). Qu and its permethylated form blocked migration and cell viability, downregulated VEGFR-2, and decreased Akt, JNK, and ERK levels on human primary endothelial cells (318). Qu altered the Akt/mTOR/AMPK/signaling (319). Qu used the anti-NSCLC effect by blocking Src-mediated Fn14/NF-κB signaling *in vitro* and *in vivo* (320). The antitumor roles of Qu generally occur *via* the modulation of VEGF, apoptosis, P13K/Akt/mTOR, MAPK/ERK1/2, and Wnt/β catenin pathways (321). Qu blocks the proteasome action *via* modulation of pathways including Akt/PI3K and ERK (322, 323). Qu was reported to suppress the melanoma and breast cancer cells by inhibiting MMP3 expression (324). Qu significantly shows anti-migratory effects (292).

### 6.6 Mechanism of Venetoclax in NSCLC

Researchers developed a successful Bcl-2 inhibitor called venetoclax (325). It inhibited Bcl-2 only and developed the same BH3 mimetic SMI (326). After showing successful results in clinical trials while treating chronic lymphocytic leukemia (CCL), venetoclax was approved by FDA as a second-line drug to treat CCL (327). It is a selective inhibitor of Bcl-2 family proteins that stimulate apoptosis in several cancers, including lung cancer. Venetoclax (ABT-199) has revealed clinical efficiency in numerous hematological tumors (328)

#### 6.6.1 Effect of Venetoclax on Bax/Bcl-2 Cascade

Venetoclax is a selective and potent inhibitor of Bcl-2. It has revealed clinical efficiency in numerous hematological tumors (328). This inhibitor continues to attach to Bcl-2 (329). Venetoclax stimulated BIM-dependent cell death *in vitro*, inhibited cancer growth, and stimulated tumor failures in mice bearing more Bcl-2–expressing SCLC cancers *in vivo*. However, venetoclax is a potential therapy for more Bcl-2-expressing SCLCs (330). SCLC displays elevated Bcl-2 expression and may be accountable to single-drug treatment *via* venetoclax. Increased Bcl-xL venetoclax explained preclinical trial action in breast tumor cells (151). Hence, venetoclax is a promising Bcl-2 inhibitor. Bcl-2 is a target in specific subtypes of human T-ALL that can be utilized by venetoclax (331). The new combination of decitabine with venetoclax was proficient and well tolerated in old AML patients (332). However, venetoclax presents the first-in-class Bcl-2 inhibitor careful platelets (333).

#### 6.6.2 Effect of Venetoclax on EGFR-Mediated Pathway

Venetoclax can work by diverse signaling pathways for accomplishing synergistic cytotoxicity with AZD9291 in NSCLC (H1975AR). Hence, this inhibitor might provide an effective choice in combination therapy with EGFR-TKIs for treating NSCLC with EGFR-TKI resistance (334). However, combining radiation for EGFR and Bcl-2 obstruction may be a new plan for targeting cancer stem cells (335). A study supports a preventive, therapeutic targeting of bioenergetics and mitochondrial primarily for impacting early drug-escape appearance using the EGFR accuracy inhibitor combined with a wide BH3 mimetic for interrupting Bcl-xL/Bcl-2 together (336). NF-κB was drastically less regulated in AZD9291+ABT-199 treatment groups than AZD9291 or ABT-199 treatment only (334). The combination of ABT-199 + irradiation + cetuximab enhanced the blockage of the 2D and 3D cell proliferation, migration, and resistance to cell death. Additionally, in a nude model with a heterotopic tumor xenograft, a treatment combining ABT-199 with fractional cetuximab irradiation delayed the cancer growth and enhanced *in vivo* lifespan without side effects (335). ABT-199 has been checked in combinations with TKIs such as imatinib, nilotinib, and dasatinib in cells with blast-crisis CML. This study revealed in a CML mouse model that ABT-199 alone or in combination is better than nilotinib in removing CML stem cells *in vivo*. However, to study the dual inhibition of Bcl-2 and Mcl-1, HHT and ABT-199 were combined and examined in seven diffuse large B-cell lymphoma cells (337).

## 7 Combined Therapeutic Strategy for EGFR-Mediated NSCLC

EGFR belongs to the tyrosine kinase family. EGFR dimerization is responsible for cell proliferation, survival, and invasion (26, 338). Inhibiting pathways by EGFR presents an excellent strategy for therapeutic interference. Gefitinib and erlotinib are selective EGFR TKIs explaining anticancer action either singly or combined with radiation therapy and chemotherapy in human cancer xenografts (339–341). Effective drugs used in EGFR-embattled therapies are erlotinib and gefitinib (121, 342), also known as EGFR TKIs, which face resistance during the treatment of advanced-stage NSCLC (95, 343, 344). This resistance is due to a mutation caused by exon 19 deletions and missense mutation on exon 21 (120, 345, 346). Until the current utilization of TKIs, the standard first-line management and treatment for patients with unresectable NSCLC and excellent presentation status have engaged the employment of combined chemotherapy with regimens (347).

Vemurafenib and dabrafenib are used in single-agent target therapy against NSCLC patients with BRAF mutation (348–351). This mutation is caused due to a single transversion at exon 15, where valine is replaced through glutamate (residue 600) (352). Hence, other potential gene targets in the case of NSCLC are HER2, NTRK, Bax, and Bcl-2 (353, 354). Cetuximab-induced targeting of EGFR stimulated tumor cell death (355, 356). The TGFα-EGFR pathway in both cancer-associated endothelial cells and cancer cells themselves is essential in the development of colon cancer. However, repealing the pathway activation *via* a double tyrosine kinase inhibitor in combined therapy may significantly reduce cancer cell proliferation and stimulate apoptosis in both cells. However, targeting the VEGFR and EGFR pathway in cancer vasculature with anti-neovascular therapy offers a new plan for colon cancer treatment. Cetuximab, an anti-EGFR antibody, is moderately effective in EGFR-expressing cells (357). The T790M mutation is a promising target for NSCLC patients (358). However, new therapies are required to conquer resistance to the drug. Crizotinib (MET inhibitor) might enhance the gefitinib susceptibility in NSCLC (359).

Numerous *in vitro* studies explained the anticancer effect and potential mechanisms of EGCG on tumor cells. The combination treatment blocked the EGFR pathway and reduced the p-EGFR, p-ERK, and p-Akt expression. EGCG and cDDP have exhibited a potential therapeutic effect in NSCLC patients (219). BIM polymorphism is strongly linked to a poor clinical reaction for EGFR TKIs in EGFR-mutant NSCLC patients; hence, BH3-mimetic ABT-737 returns BIM functionality EGFR-TKI sensitivity (232). ABT-737 drastically increases erlotinib-mediated cell death, and more strong responses for EGFR inhibitors in lung tumor patients harbor EGFR kinase domain mutations (233). TQ is associated with many pathways and stimulated apoptosis in tumor cells *via* inhibiting the STAT3 pathway by inhibiting JAK2- and Src-induced phosphorylation of EGFR-TK (253, 284, 288). However, Qu displayed potent cytotoxic results on NSCLC cells harboring the EGFR C797S mutation *via* preventing AXL and stimulating cell death (316). Venetoclax may work by synergistic cytotoxicity with AZD9291 in NSCLC (H1975AR). Hence, this inhibitor might provide a productive choice in combination therapy with EGFR-TKIs for treating NSCLC with EGFR-TKI resistance (334). Therefore, several therapeutic strategies to target the EGFR pathway demonstrated various efficiencies that overcome drug resistance and cancer development.

## 8 Conclusions and Future Prospects

The EGFR pathway is associated with several cancer progressions, including NSCLC. The EGFR pathway regulates Bax/Bcl-2 cascade in NSCLC. Inhibition of EGFR leads to upregulation of pro-apoptotic proteins and stimulates apoptosis by activating the intrinsic apoptotic pathway. Targeted therapy might finally alter the treatment model for lung cancer and provide an expectation for patients with inadequate treatment opportunities. New targeted therapies offer a novel hope for cancer patients, including NSCLC, a rare disease for standard treatments. In the last decades, the improvement in cellular, molecular, and cancer biology research could be distinct by some foundational pillars—one of the most significant ones being the beginning of SMIs/phytochemicals. Targeting the EGFR with SMIs is a suitable validated strategy in tumor therapy. EGFR SMIs have been approved worldwide for the treatment of multiple cancers. However, these drugs explained high efficiency in cancer therapy.

Several clinical trials for the SMIs/agents of targeted cancer therapy are ongoing and have illustrated potent and promising effects to date. Hence, these trials assist in describing the function of targeted cancer therapy in the management and treatment of tumors, including NSCLC. Therefore, the dispute for the clinical improvement and utilization in cancer therapy of anti-EGFR agents alone and/or in combination with other SMIs/phytochemicals treatments would be the suitable assortment of potentially responding NSCLC patients. In the future, combined therapies with molecular mechanisms might lead to the eventual therapeutic option. However, targeting EGFR-mediated Bax/Bcl-2 cascade would be a potential therapy for NSCLC. In prospect studies, this study should significantly assist in the approach of new inhibitors for the EGFR-mediated Bax/Bcl-2 cascade that facilitate the treatment and management of NSCLC. Additionally, a close collaboration between molecular biologists, clinicians, and pathologists is critical for developing target therapy for NSCLC.

## Author Contributions

MaA: conceptualization, writing—original draft preparation, data curation, investigation, methodology. SA: writing—original draft preparation, data analysis, validation, visualization. MoA: formal analysis, writing—review and editing, investigation, and validation. AME: data curation validation, writing—review and editing. WA: methodology, writing—review and editing. MAl: investigation, validation, writing—review and editing. YH: investigation, validation, writing—review and editing. AT: data curation, writing—review and editing. AS: data curation, writing—review and editing. VIP: conceptualization, data analysis, validation, project administration, writing—review and editing. MIH: conceptualization, investigation, writing—original draft preparation, review and editing.

## Funding

This work is supported and funded through the Indian Council of Medical Research (Grant No. 45/6/2020-DDI/BMS).

## Conflict of Interest

The authors declare that the research was conducted in the absence of any commercial or financial relationships that could be construed as a potential conflict of interest.

## Publisher’s Note

All claims expressed in this article are solely those of the authors and do not necessarily represent those of their affiliated organizations, or those of the publisher, the editors and the reviewers. Any product that may be evaluated in this article, or claim that may be made by its manufacturer, is not guaranteed or endorsed by the publisher.

## Abbreviations

Bcl-2, B cell lymphoma 2; OSCC, oral squamous cell carcinoma; MMP, mitochondrial membrane permeabilization; MMP mitochondrial membrane potential; CLL, chronic lymphocytic leukemia; BH, Bcl-2 homology; NSCLC, non-small cell lung carcinoma/cancer; LUSC, lung squamous cell carcinoma; PKB, Akt/protein kinase B; NFκB, nuclear factor κB; JAK2, Janus-activated kinase-2; EGFR, epidermal growth factor receptor; TNFR, tumor necrosis factor receptor; MAPK, mitogen-activated protein kinase; ERK1/2, extracellular signal-regulated kinase1/2; JNK, c-Jun N-terminal kinase; AP-1, activator protein-1; PARP, poly-(ADP-ribose) polymerase; TQ-I3M, TQ–indirubin-3-monoxime; IDEAL, Iressa Dose Evaluation in Advanced Lung Cancer; TKIs, tyrosine kinase inhibitors; NTRK, neurotrophic tyrosine receptor kinase; STAT3, signal transducer and activator of transcription-3; EGCG, epigallocatechin-3-gallate.
